# Latent profiles of parenting concerns among young and middle-aged cancer patients: A cross-sectional study

**DOI:** 10.1097/MD.0000000000045523

**Published:** 2025-11-21

**Authors:** Yan-Mei Gu, Jie Yu

**Affiliations:** a Oncology Department, Affiliated Hospital of Nantong University, Nantong, Jiangsu, PR China; b Interventional Radiology and Vascular Surgery Department, Affiliated Hospital of Nantong University, Nantong, Jiangsu, China.

**Keywords:** influencing factors, latent profile analysis, parenting concerns, young and middle-aged cancer patients

## Abstract

Parenting concerns, as a negative emotion, is prevalent among young and middle-aged cancer patients with minor children, significantly impacting their normal cancer treatment. However, quantitative studies on the current status and influencing factors of parenting concerns in young and middle-aged cancer patients are relatively rare. This paper aims to explore the latent categories and influencing factors of parenting concerns in young and middle-aged cancer patients, thus providing a theoretical guidance for implementing targeted intervention measures in the patients. From October 2022 to June 2023, a total of 211 young and middle-aged cancer patients hospitalized in a tertiary-level hospital in Jiangsu Province were selected as the study subjects by using a convenience sampling method. The Chinese version of Parenting Concerns Questionnaire, Social Support Rating Scale, and Hospital Anxiety and Depression Scale were used for investigations. A latent profile analysis of parenting concerns in young and middle-aged cancer patients were conducted, and different categories of influencing factors were analyzed by using multivariate logistic regression analysis. The parenting concerns of young and middle-aged cancer patients were classified into 3 latent categories: low parenting concern (group A), medium parenting concern -being concerned about their partners (group B), and high parenting concern -being concerned about their children (group C). Logistic regression analysis showed that compared with group A, the number of children, subjective support and depression were risking factors to group B (all *P* < .05). The number of children, family percapita monthly income, objective support, and depression were risking factors to group C (all *P* < .05). There are obvious categorical characteristics of parenting concerns in young and middle-aged cancer patients. Healthcare professionals should provide psychological counseling and targeted intervention measures to patients according to the influencing factors of different categories to reduce their parenting concern levels and improve their qualities of life.

## 1. Introduction

According to the Global Cancer Statistics 2022,^[1]^ there will be about 20 million new cancer cases worldwide in 2024, and the incidence of cancer among young and middle-aged people (18–59 years old) shows an overall rising trend. People in this age group generally have important responsibilities in the family and society, as reported in a foreign study.^[2]^ Approximately 14% to 25% of cancer patients have minor children, and the majority of them are young to middle-aged people. Compared to patients without minor children, this group of people is not only concerned about their own illness, but also concerned about the conflict between treatment and fulfilling the responsibilities of a parent. This pressure-related emotional state caused by the inability to balance parenting and medical treatment is called parenting concern.^[3]^ It has been reported that the parenting concerns of young and middle-aged cancer patients in China is generally at the medium level or above,^[4,5]^ which has different degrees of negative impact on the normal anti-cancer treatment and physical and mental health of patients. It is important for clinical nursing workers to explore the influencing factors of parenting concerns in young and middle-aged cancer patients and implement targeted intervention measures. However, at present, most of the studies on parenting concerns at home and abroad focus on qualitative studies or scale evaluation of parenting concern level, which lack individual-specific considerations.^[6,7]^The latent profile analysis (LPA) can achieve local independence by explaining associations between exogenous variables using latent categorical variables with an individual focus.^[8]^Therefore, this study aimed to explore the categories of parenting concerns in young and middle-aged cancer patients using LPA, and investigate the heterogeneity and influencing factors of parenting concerns in young and middle-aged cancer patients, thus providing a reference for implementing targeted intervention measures in clinical practice.

## 2. Materials and methods

### 2.1. Participants

The young and middle-aged cancer patients, who were hospitalized in the oncology department of a tertiary-level hospital in Jiangsu Province from October 2022 to June 2023, were selected as the study subjects using a convenience sampling method after a cross-sectional survey. Inclusion criteria: patients aged 18 to 59 years; patients diagnosed with malignant tumors by histopathological examination, who were aware of their own condition; patients who were raising minor children aged < 18 years; and patients who were conscious and able to communicate without any difficulty, and voluntarily participated in this study. Exclusion criteria: patients who were critically ill and unable to cooperate. There was a total of 18 variables in this study, the sample size should be 5 to 10 times the number of variables,and it was calculated as 90 to 180 patients. Considering the 20% lost follow-up rate and sampling error, the sample size was expanded to 108 to 216 patients, and finally 211 patients were included in the study. This study was approved by the Ethics Committee of the Affiliated Hospital of Nantong University (2020-K079),and all patients were well informed and voluntarily participated in this study.

### 2.2. Survey instruments

We used 4 scales to assess the level of parenting concerns and related factors in middle-aged and young cancer patients. These 4 scales included General information questionnaire,Chinese Version of Parenting Concerns Questionnaire (PCQ), Social Support Rating Scale (SSRS) and Hospital Anxiety and Depression Scale (HADS). General information questionnaire was created basing on a large number of literature reviews and group discussions, which includes information such as gender, age, educational level, number of children, and child age, and the disease-related information such as time of disease diagnosis, type of disease, and clinical stage. The PCQ scale was compiled by Muriel et al,^[9]^ and Tingting Kang^[10]^ completed the Chinese translation and the reliability and validity tests of this scale in 2020. This scale consisted of 15 items in 3 dimensions, including items concerning the emotional impact on the children (item 2, 4, 7, 9, and 11), items concerning the actual impact on the children (item 1, 3, 5, 8, and 10), and items concerning the child’s father/mother (item 6, 12, 13, 14, and 15), and the items were assessed using a Likert 5-point scale, the scores ranged from 1 to 5, with 1 indicating no concern and 5 indicating extreme concern. The total score and the score for each dimension were calculated by taking their average scores of all items respectively, with a higher total score indicating a higher parenting concern level in cancer patients. The Cronbach alpha coefficient for this scale is 0.850. The SSRS was compiled by the Chinese scholar Xiao Shuiyuan.^[11]^The scale has a total of 10 items in 3 dimensions, and 3 dimensions are objective support (item 2, 6, and 7), subjective support (item 1, 3, 4, and 5), and utilization of social support (item 8, 9, and 10). The objective support is the material support provided by others; the subjective support is the emotional support provided by others; and the utilization of social support represents the individual’s acceptance of the support provided by others. The Cronbach alpha coefficient for this scale is 0.890.The HADS^[12]^ was developed by Zigmond and Snaith in 1983. This scale is used to screen for anxiety and depression among general hospital patients. The scale is divided into 2 subscales: anxiety and depression, with 14 items and scores ranging from 0 to 21. A higher score indicates a more severe level of anxiety and depression. Evaluation criteria: 8–10 points indicate mild anxiety and depression, 11–15 points indicate moderate anxiety and depression, and more than 15 points indicate severe anxiety and depression. This scale is simple and easy to administer, and has been widely used in clinical practice. The Cronbach alpha coefficient of this scale is 0.711.

### 2.3. Data collection and quality control methods

The data collection was carried out by 3 postgraduate students and 2 clinical nurses in our department, and the investigators received unified training, and then conducted one-on-one surveys with the study subjects. The specific survey process was as follows: the investigators explained the purpose and content of the survey to the study subjects; the patients signed a informed consent form, and then filled out the survey form. For the questions raised by the patients during the survey process, relevant answers were given to the patients on the basis of full respect for the patients’ right to make their own choices. The missing items were asked again by the investigator to ensure the completeness of the questionnaire survey. A total of 220 questionnaires were distributed in this study, and 211 valid questionnaires were recovered, with a valid recovery rate of 95.6%.

### 2.4. Statistical analysis

The latent profile model was established by using Mplus8.0, and the 3 dimension scores in the PCQ scale were used as exogenous variables to carry out the latent profile analysis, and the latent categories of the parenting concerns in young and middle-aged cancer patients were analyzed. At first, it was assumed that there was only one category, and then the number of categories was incrementally increased, finally the best model was selected according to fitting indicators by comprehensive analysis of the parameters of various models. The classification of LPA mainly referred to 3 types of fitting indicators:^[13]^model fitting indicators -Akaike information criterion, Bayesian information criterion (BIC) and sample-size adjusted BIC (a BIC), the smaller the above values were, the better the fitting effect of the model was; the classification indicator was entropy, its value range was 0 to 1, a value closer to 1 indicated a higher accuracy; the indicators of likelihood ratio test are composed of 2 indicators such as LoMendell-Rubin likelihood ratio test (LMR-LRT) and Bootstrap likelihood ratio test (BLRT). When *P* < .05, the model of category k was superior to that of category k-1.

SPSS 25.0 was used for statistical analysis. The measurement data conforming to normal distribution were expressed as mean ± standard deviation, and the independent samples t-test was performed for comparison among groups; the measurement data not conforming to normal distribution were expressed as medians (25th percentile, 75th percentile); the counting data were expressed as frequency and percentage. The classification results of LAP were used as the dependent variables, and the general data with statistical significance were screened as the independent variables by conducting comparison among groups. Multiple logistic regression analysis were performed used to explore the influencing factors for different categories of parenting concerns among young and middle-aged cancer patients, and *P* < .05 was considered to be statistically significant.

## 3. Results

### 3.1. General data and current status of parenting concerns among young and middle-aged cancer patients

Of the 211 cancer patients, 64 were males and 147 were females. The total average score of parenting concerns was 2.87 ± 0.36 points, and the average score range in each dimension was 2.57 to 3.24 points. Other general data are presented in Table 1.

**Table 1:** Univariate analysis of latent categories of parenting concerns in young and middle-aged cancer patients (n = 211).

Items		Category 1	Category 2	Category 3	Statistical value	*P* value
Number of children (cases, %)	1 child	39 (75.0)	5 (9.6)	8 (15.4)	12.764^*^	.002
	2 children	75 (47.2)	44 (27.7)	40 (25.2)		
Family percapita monthly income(cases, %)	<2000 Yuan	1 (25.0)	2 (50.0)	1 (25.0)	12.0152^†^	.044
2000–4000 Yuan		22 (46.8)	17 (36.2)	8 (17.0)		
4000–6000 Yuan		40 (60.6)	15 (22.7)	11 (16.7)		
	>6000 Yuan	51 (54.3)	15 (16.0)	28 (29.8)		
Time of disease diagnosis (cases, %)	<1 mo	13 (52.0)	12 (48.0)	0	17.706^*^	.007
	1–6 mo	58 (57.4)	19 (18.8)	24 (23.8)		
	6–12 mo	22 (61.1)	6 (16.7)	8 (22.2)		
	>12 mo	21 (42.9)	12 (24.5)	16 (32.7)		
Social support score ($\bar{x}\pm s$)	Objective support	11.22 ± 2.64	9.84 ± 2.39	9.69 ± 2.05	6.869^‡^	.001
Subjective support		22.58 ± 5.83	19.27 ± 4.99	20.73 ± 4.71	9.2033^‡^	<.001
Utilization of social support		7.06 ± 2.20	6.14 ± 2.03	6.29 ± 2.11	4.1213^‡^	.018
Depressionscore (points, $\bar{x}\pm s$)		13.52 ± 2.13	12.67 ± 2.44	13.17 ± 1.88	7.4263^‡^	.001

* χ^2^.

† Fisher exact probability method.

‡ *F*-value.

### 3.2. Results of latent profile analysis of parenting concerns in young and middle-aged cancer patients

A latent profile analysis of parenting concerns in young and middle-aged cancer patients was performed, starting from profile 1 and increasing sequentially to profile 4, as shown in Table 2. When fitting category 3, the entropy value was 0.821, which was better than the dichotomous classification, and its P(LMRT) and P(BLRT) were both <0.05, and when fitting category 4, the P(LMRT) and P(BLRT) were both >0.05, so that the 3 categories were selected as the optimal model.

**Table 2:** Latent profile analysis models of parenting concerns in young and middle-aged cancer patients (n = 211).

Model	AIC	BIC	aBIC	Entropy	*P* value		Category probability
					P(LMR-LRT)	P(BLRT)	
1	1038.624	1058.735	1039.723	——	——	——	——
2	991.289	1024.808	993.121	0.809	.0001	<.001	0.647, 0.353
3	973.705	1020.631	976.270	0.821	.0088	<.001	0.527, 0.219, 0.254
4	979.133	1039.467	982.432	0.788	.5989	1.000	0.597, 0.144, 0.183, 0.0759

aBIC = sample-size adjusted Bayesian information criterion, AIC = Akaike information criterion, BIC = Bayesian information criterion, BLRT = Bootstrap likelihood ratio test, LMR-LRT = LoMendell-Rubin likelihood ratio test

### 3.3. Naming of latent categories of parenting concerns in young and middle-aged cancer patients

**Figure 1.**
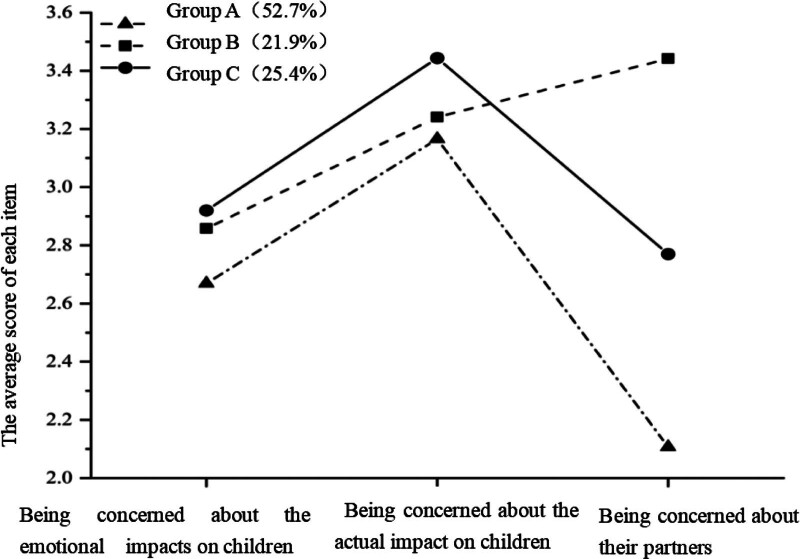
Distribution of characteristics of 3 latent categories of parenting concerns in young and middle-aged cancer patients. Group A contained 52.7% patients with low parenting concern who were most concerned about the actual impact on children; group B contained 21.9% patients with medium parenting concern who were most concerned about their partners; group C contained 25.4% patients with high parenting concern, who were most concerned about the emotional impacts on children.

There were 114 patients (52.7%) in category 1, who had the lowest total score in parenting concerns were named “low parenting concern(group A)”; there were 49 patients (21.9%) in category 2, with scores at a medium level, and were therefore named “medium parenting concern -being concerned about their partners (group B)”; there were 48 patients (25.4%) in category 3, who had the highest scores for being concerned about the emotional impacts on the children, and were therefore named “high parenting concern -being concerned about the impacts on children(group C),” as shown in Figure 1.

### 3.4. Univariate analysis of latent categories of parenting concerns in young and middle-aged cancer patients

There were statistically significant differences in the number of children, family percapita monthly income, time of disease diagnosis, social support, and depression among the 3 latent categories of parenting concerns in young and middle-aged cancer patients (all *P* < .05), while there were no statistically significant differences in others, and the items with statistically significant differences were shown in Table 1.

### 3.5. Multifactorial analysis of latent categories of parenting concerns in young and middle-aged cancer patients

A logistics regression analysis was conducted with the parenting concerns in young and middle-aged cancer patients as the dependent variables, and the indicators with statistically significant differences in the univariate analysis as the independent variables, with category 1 as the control; the number of children, subjective support and depression were risking factors to group B (all *P* < .05). The number of children, family percapita monthly income, objective support, and depression were risking factors to group C (all *P* < .05).The independent variable assignments were shown in Table 3, and the results were displayed in Table 4.

**Table 3:** Independent variable assignment.

Independent variable	Assignment method
Number of children	1 = 1;≥2 = 0
Family percapita monthly income (yuan)	<2000 = (0,0,0); 2000–4000=(1,0,0); 4000–6000 = (0,1,0);>6000=(0,0,1)
Time of disease diagnosis	<1 mo = (0,0,0); 1–6 mo = (1,0,0); 6–12 mo = (0,1,0); more than 12 mo = (0,0,1)
Social support	The original values were directly analyzed.
Depression	The original values were directly analyzed.

**Table 4:** Multiple logistic regression analyses of latent categories of parenting concerns in young and middle-aged cancer patients.

Items	Group B (compared with group A)				Group C (compared with group A)			
	β	*P*	OR	95%CI	β	*P*	OR	95%CI
Constant term	−0.121	.941	—	—				
Number of children								
1	1.439	.008	1.237	1.082–1.686	−0.804	.080	0.447	0.182–1.102
Family percapita monthly income (yuan)								
<2000	0.883	.507	2.418	0.178–32.832	−0.346	.817	0.708	0.038–13.214
2000–4000	0.711	.147	2.035	0.779–5.320	−0.589	.246	0.555	0.205–1.502
4000–6000	−0.231	.619	0.794	0.320–1.969	−0.950	.031	0.387	0.163–0.919
Social support								
Subjective support	−0.082	.039	0.921	0.852–0.996	−0.028	.467	0.972	0.902–1.048
Objective support	−0.019	.844	0.982	0.815–1.182	−0.199	.025	0.820	0.689–0.975
Utilization of social support	−0.094	.345	0.910	0.748–1.107	−0.082	.401	0.922	0.762–1.115
Depression	0.223	.039	1.249	1.011–1.544	0	.997	1.000	0.806–1.243

Group A was used as control.

## 4. Discussion

### 4.1. Current status of parenting in among young and middle-aged cancer patients

In this study, the total score of parenting concerns in young and middle-aged cancer patients was 2.87 ± 0.36, with the highest average score of 3.24 ± 0.53 in the dimension of being concerned about the actual impact on children, which was at a medium-high level. The total score and the average score in each dimension were lower than those in the study by Kang Tingting^[4]^ in China, and the reason may be that the geographical location of study subjects were far apart from each other, and there were great differences in parenting ideas, economic level and social support among them. Meanwhile, the total score and the average score in each dimension were higher than the scores in the study by Park EM^[14]^ in America. Chinese Confucianism, with an isomorphism between the family and the state as its ethical foundation, considers the family as the basic unit of society and takes family as the core,^[15]^ “It is the fault of a father not to educate his children,” this well-known saying clearly points out the inescapable responsibility of parents in educating their children.Under the influence of this culture, the personal values of family members are often realized through assuming family responsibilities. Therefore, Chinese parents have a stronger sense of responsibility in raising their children. If the diseases weaken their abilities to raise their children, this will intensify their concerns about parenting. Unlike a family-oriented mindset in Chinese Confucian ideology, the Western concept of the family, refined by the Renaissance and the Enlightenment, has developed a value orientation based on individualism, with a greater emphasis on the independence and autonomy of the individuals, and the concepts of child-rearing naturally form a sharp contrast with those of China. Therefore, medical personnel in China should pay more attention to the status of parenting concerns in young and middle-aged cancer patients who are raising minor children, and provide timely and appropriate intervention measures.

### 4.2. The parenting concerns in young and middle-aged cancer patients exhibit significant heterogeneity, and can be classified into 3 categories

The results of this study showed that the parenting concerns in young and middle-aged cancer patients had obvious classification characteristics, which were classified into 3 categories such as group A, B, and C. Patients in group A generally manage these concerns better. They may benefit from a robust social support network and effective symptom management. These patients often demonstrate strong psychological resilience and maintain a positive, optimistic attitude toward their role as parents. Patients in group B (those with more intense concerns about their partners) exhibit stronger worries about how cancer affects their partners. Driven by awareness of cancer’s impact on family roles, these individuals not only worry about their own health but also focus on how the disease might affect their partners and children.After deep communications with these patients we found that they usually played the role of a “good wife” or “good husband” in the family, not only taking care of their children, but also arranging and taking care of their partners’ daily lives, so that they can work at ease outside.Therefore, after being diagnosed with cancer, the patients are also worried that their partners’ daily lives will be affected. For such patients, we suggest that we can arrange for their partners to come to the hospital and organize a lecture for them to alleviate patients’ concerns, so that they can understand their partners’ worries about them and share the daily chores that the patients have to do for them as soon as possible,and the patients can stop worrying about them, get proper treatment and recuperate without any worries. Patients in group C (those with severe parenting concerns) had the highest level of concerns about their children,.the results were consistent with the findings of Liu et al.**^[16]^** The reason may be that more than half of the children in this study were under the age of 13 years and had not enter the puberty period, and their abilities to independently arrange daily life were still insufficient, and their daily lives relied on the helps of their parents; however, sudden diseases and frequent hospitalizations disrupted the previous regular lives, thus the parents would be concerned about the effects of their own diseases on their children’s actual lives. It is suggested that the medical personnel should pay attention to cancer-affected families with minor children. It is advisable to encourage the patient’s spouse or other family members to jointly participate in the care of the minor children who are still young. The minor children who have entered the upper grades of primary school or junior high school can be helped to develop independent living habits, learn to plan their lives outside of study without relying on their parents, and thus grow up to be a qualified “little adult” as soon as possible. Through the participation and efforts of the whole family, the patients can eliminate their worries about their children’s actual lives, pay active attention to their own health and cooperate with doctors to complete the treatments.

### 4.3. Differences in the distribution of parenting concerns in young and middle-aged cancer patients

Patients with 2 children and a low income level are more likely to belong to group C.

The results of this study showed that the patients with a greater number of children had a higher possibility of belonging to group C (*P* = .008, OR = 1.439, 95% CI [1.082–1.686]), which were consistent with the results of Hao Limin et al.^[5]^ Compared with group A, the patients with a low income level had a higher possibility of belonging to group C (*P* = .031, OR = 0.387, 95% CI [0.163–0.919]), and the reason may be that the cancer patients raising 2 minor children need to bear more childcare burdens and these burdens mainly come from 2 aspects: energy and finance. Moreover, the minor children have physical, psychological and social immaturity, with a higher dependence on their parents, and the cancers have weakened the roles of the patients as a father or a mother, thus the patients cannot provide care and emotional supports for their children as they used to do, especially those with 2 children or a low income level, which aggravates the contradiction and increases the patient’s concern about their children. Therefore, clinical healthcare workers should pay special attention to cancer patients with 2 minor children or poor family economic conditions, and provide personalized support measures for patients through fully understanding the patients and their children. For example, the childcare training courses can be offered.for the cancer patients with younger children, and their parenting confidence can be enhanced by improving their parenting skills; a comfortable environment can be provided for cancer patients with adolescent children to create a good communication condition for both patients and their children, encourage open communication between them, even directly discuss the issue of death, and offer corresponding solutions to their individual current predicaments, so as to release negative emotions from both sides; the physicians need to take into account the efficacy and cost of treatment to formulate a reasonable treatment plan for cancer patients with low family incomes, so as to reduce their financial burdens, lower their levels of parenting concerns, and enable them to actively cooperate with treatment.

### 4.4. Patients with less subjective and objective supports are more likely to belong to group C

The results of this study showed that the patients with less subjective and objective supports had a higher possibility of belonging to group C (*P* = .039, OR = 0.921, 95% CI [0.852–0.996]), which is in line with that in the study by Laura et al.^[17]^ The reason for this may be that in addition to coping with the burden of their own illnesses, the young and middle-aged cancer patients are particularly concerned about the disruption of family life due to frequent hospitalization, which may have different degrees of impact on the study and life of children. If the social support level is insufficient, the patients may be overly concerned about the lives of their minor children, which may affect their treatment decision-making and adherence to treatment.^[18]^Therefore, it is recommended that the medical personnel should fully understand the social support level for young and middle-aged cancer patients, and pay more attention to those with less subjective and objective supports. At the hospital level, some family and child-friendly rooms can be provided for patients to spend this difficult time with their children while being treated; the department can arrange relevant psychological counselors to provide open consultation hours to address patients’ psychological problems and confusion in a timely manner; the communication should be provided to family members of patients with insufficient family support, emphasizing the importance of family support in lowering the level of parenting concerns, and encouraging other family members such as their partners, parents, or other relatives and friends to participate in taking care of the children’s daily lives during school time to ensure they can focus on their studies, so as to make the patients feel comfortable and at ease while receiving treatment.^[19]^

### 4.5. Patients with a higher level of depression are more likely to belong to group C

Multiple logistic regression analyses showed that the patients with higher depression scores had a higher possibility of belonging to group C (*P* = .035, OR = 1.249, OR [1.011–1.544]), this finding is consistent with that of Kathrin Milbury et al.^[20]^The reason may be that they have poorer psychological adjustment ability than these patients who are positive and optimistic. Depression, as a negative mood characterized by low mood and loss of interest, can seriously affect the treatment outcome and quality of life in patients. Previous studies have pointed out^[21]^ that the quality of life is significantly negatively correlated with the level of parenting concerns in patients, that is, the lower quality of life eventually lead to an increasing level of parenting concerns. Therefore, it is recommended that the nursing staff can identify depressed patients at an early stage, and actively guide patients to learn to release negative emotions such as anxiety and depression by conducting lectures on emotion management in cancer patients. At the same time, the nursing staff can stimulate patients’ intrinsic motivation by sharing the beautiful moments they have with their children, and promote the development of patients’ intrinsic self-regulation ability while satisfying their basic inner needs, so as to improve their overall mental health, help them get rid of the worries about their children’s emotions and adopt a positive attitude towards the treatment.

## 5. Limitations and strengths

Most current studies on parenting concerns are mainly qualitative studies, this study used multiple scales to systematically and comprehensively analyze the influencing factors of parenting concerns in young and middle-aged cancer patients from multiple dimensions such as patient personal data, disease-related factors, anxiety and depression levels, and social support rating. Moreover, this study used Mplus8.0 software to perform potential profile analysis, achieved precise classification of patients and paid more attention to heterogeneity of individuals, providing a scientific basis for offering more precise and targeted nursing measures in clinical practice.

However, some limitations must be acknowledged. This study was performed in a single tertiary hospital, and the sample selection was limited,which could not represent the overall parenting concern level of Chinese cancer patients. In the later stage, a multi-center, large-sample longitudinal study will be conducted to further validate and supplement the results of this study. In addition, this study was a cross-sectional survey, which failed to investigate the changes in the parenting concern level of patients over time, thus the longitudinal study can be conducted to explore the longitudinal trajectory of the parenting concern level in the future.

## 6. Conclusions

The parenting concerns in young and middle-aged cancer patients can be classified into 3 categories such as low parenting concern, being concerned about the actual impact on children, medium parenting concern, being concerned about their partners, and high parenting concern-being concerned about the emotional and actual impacts on children. The number of children, Family percapita monthly income, subjective support, objective support, and depression are the influencing factors of different categories of parenting concerns, and the medical professionals should provide individualized and targeted intervention measures based on different categories and characteristics.

## Acknowledgments

The authors gratefully acknowledge the nursing administrators in the enrolled hospitals for permitting and facilitating the investigation, and all 211 participants for responding to the survey.

## Author contributions

**Conceptualization:** Jie Yu.

**Investigation:** Yan-Mei Gu, Jie Yu.

**Methodology:** Jie Yu.

**Project administration:** Jie Yu.

**Writing – original draft:** Yan-Mei Gu.

**Writing – review & editing:** Yan-Mei Gu.
